# Optimizing fodder yield and quality through integrated organic nutrient amendments in multi-crop system

**DOI:** 10.3389/fpls.2025.1517399

**Published:** 2025-02-12

**Authors:** Santosh Onte, Vrushabh Vijay Fiskey, Manjunath S. Melavanki, Airadevi P. Angadi, Prasanna S. Pyati, Magan Singh, Dileep Kumar, Sanjivkumar Angadarao Kochewad, Sudhir Kumar, Hari Om, Vijendra Kumar Meena, Kamal Garg, Vetrivel Karunakaran, Manish Kanwat, Babu Lal Meena, Yogananda Shivalli Boregowda, Rahul Bellagi, K. Naveena, Sunil Chandersheker, Elisa Azura Azman, Sanjeev Kumar

**Affiliations:** ^1^ ICAR-Krishi Vigyan Kendra, University Agricultural Sciences, Dharwad, Karnataka, India; ^2^ Land and Water Management Research Group, Centre for Water Resources Development and Management (CWRDM), Calicut, India; ^3^ Agronomy Section, ICAR-National Dairy Research Institute, Karnal, Haryana, India; ^4^ Division of Agronomy, Tocklai Tea Research Institute, Jorhat, Assam, India; ^5^ Division of Crop Production, ICAR-Indian Institute of Sugarcane Research, Lucknow, Uttar Pradesh, India; ^6^ School of Edaphic Stress Management, ICAR- National Institute of Abiotic Stress Management, Baramati, Maharashtra, India; ^7^ Division of Plant Physiology, ICAR-Indian Agricultural Research Institute, New Delhi, India; ^8^ Department of Agronomy, Bihar Agricultural University, Bhagalpur, Bihar, India; ^9^ ICAR- Krishi Vigyan Kendra, Thiruvarur, Tamil Nadu, India; ^10^ ICAR-CAZRI Krishi Vigyan Kendra, Gujarat, India; ^11^ Project Coordinating Unit, ICAR- Central Soil Salinity Research Institute, Karnal, India; ^12^ College of Agriculture, Vishweshwaraiah Canal Farm, Mandya, Karnataka, India; ^13^ Indian Council of Social Science Research, Centre for Multi-disciplinary Development Research, Dharwad, Karnataka, India; ^14^ Department of Agronomy, Keladi Shivappa Nayaka University of Agricultural and Horticultural Sciences, Shivamogga, Karnataka, India; ^15^ Department of Crop Science, Faculty of Agriculture, Universiti Putra Malaysia, Serdang, Malaysia

**Keywords:** farmyard manure, organic, panchagavya, PGPR, proximate analysis, quality, yield

## Abstract

The increasing demand for organic fodder has highlighted the need for sustainable agricultural practices that optimize both yield and quality. However, research on integrated organic nutrient management in multi-crop systems remains limited, especially concerning its impact on forage productivity and nutritive value. This study aims to investigate the effects of integrated organic nutrient amendments on the yield and quality of multi-crop system. Conducted during the period 2018 to 2021 using a randomized complete block design, the experiment encompassed seven treatments, each replicated three times. These treatments comprised of different combination of farmyard manure (FYM), Plant growth promoting rhizobacteria (PGPR) and foliar spray of panchagavya for maize (M), berseem (B), and cowpea (C) and a treatment with recommended dose of fertilizers. The treatment includes T_1_: 100% RDN through FYM (M) - No application (B) - No application (C); T_2_: 50% RDN through FYM + PGPR + 3% foliar spray of panchagavya (M) – PGPR + 3% foliar spray of panchagavya (B) – PGPR + 3% foliar spray of panchagavya (C); T_3_: 75% RDN through FYM + PGPR (M) - PGPR (B)- PGPR (C); T_4_: 100% RDN through FYM + PGPR (M) - PGPR (B) - PGPR (C); T_5_: 75% RDN through FYM + PGPR + 3% foliar spray of panchagavya (M) – PGPR + 3% foliar spray of panchagavya (B) – PGPR + 3% foliar spray of panchagavya (C); T_6_: 100% RDN through FYM + PGPR + 3% foliar spray of panchagavya (M) – PGPR + 3% foliar spray of panchagavya (B) – PGPR + 3% foliar spray of panchagavya (C), and T_7_: 100% RDF (M)- 100% RDF (B)- 100% RDF (C). Research findings revealed that the T_6_ treatment, involving T6: 100% RDN through FYM + PGPR + 3% foliar spray of panchagavya (M) – PGPR + 3% foliar spray of panchagavya (B) – PGPR + 3% foliar spray of panchagavya (C), yielded significant improvements in both green fodder of maize (35.4, 37.0, and 38.6 t ha^-1^), berseem (58.2, 60.0, and 60.6 t ha^-1^) and cowpea (25.7, 27.5, and 28.3 t ha^-1^) during 2018-19, 2019-20 and 2020-21, respectively. Furthermore, T_6_ significantly enhanced forage quality, as evidenced by higher crude protein (6.4–14.8%), ether extract (19.2–40.1%), and total ash (6.5–22.1%) contents, coupled with reductions in fiber components. These findings highlight the effectiveness of integrated organic nutrient amendments in enhancing both yield and quality, offering a sustainable strategy to improve livestock feed and promote environmentally friendly agricultural practices.

## Introduction

Increasing awareness of health and nutritional needs has led to a rising demand for organic milk, recognized for its health benefits. A significant challenge in meeting this demand is the limited availability of high-quality organic fodder (Meena et al., 2018; Vaarst et al., 2019). Ensuring both high forage productivity and superior nutritive value is essential for supporting the organic livestock industry. Fodder quality, including aspects such as fiber content, protein levels, digestibility, and nutrient balance, plays a crucial role in supporting the nutritional needs of livestock and improving milk quality. However, current nutrient management practices often fall short in meeting these needs, impeding sustainability and profitability. While organic farming has a growing emphasis on sustainability, the quality of organic fodder is often compromised by insufficient nutrient management, particularly in terms of key elements that influence livestock health, like nitrogen, phosphorus, and potassium levels. Adequate nutrient management is vital not only for yield improvement but also for enhancing the fodder’s nutritional value (Escribano, 2018; Timsina, 2018). This gap presents obstacles to both the sustainability and profitability of organic livestock farming, making it essential to address these challenges to support the burgeoning organic dairy industry (Odhong et al., 2014; Wolde and Tamir, 2016).

Maize, berseem, and cowpea are essential crops for food and fodder production, with varying production statuses depending on region and environmental conditions. Maize is one of the most widely grown cereal crops globally, primarily cultivated in North America, Latin America, Asia, and Africa, with significant production in the U.S., China, Brazil, and India. Berseem, a popular forage legume, is primarily grown in South Asia and the Mediterranean region, serving as both fodder and green manure. Cowpea, grown mainly in sub-Saharan Africa, South Asia, and parts of North America, is valued for its drought resilience and high-protein seeds. In the Indian subcontinent, maize, berseem, and cowpea are vital fodder crops that provide essential energy, protein, and carbohydrates necessary for livestock health. These crops are characterized by high nutritional value, contributing significantly to livestock productivity. While maize is nutrient-intensive and can deplete soil fertility (Mathur et al., 2023; Teressa et al., 2024), berseem and cowpea contribute to soil health through nitrogen fixation and nutrient solubilization, improving soil fertility for subsequent crops (Meena and Lal, 2018). These crops form a vital component of sustainable livestock feeding systems, enhancing productivity while promoting soil conservation (Das et al., 2018; Meena and Lal, 2018).

Organic amendments such as FYM, PGPR, and panchagavya hold immense potential for optimizing crop productivity and improving forage quality. These supplies essential nutrients like nitrogen, phosphorus, and potassium in a slow-release form, fostering healthy plant growth (Aytenew and Bore, 2020; Khayat, 2021). When combined with other organic inputs like green manure, compost, and biofertilizers, FYM improves nutrient diversity and soil fertility. Its role in promoting microbial activity and suppressing soil-borne diseases makes it crucial for sustainable fodder production (Verma et al., 2020; Bhunia et al., 2021). Furthermore, studies have shown that the use of organic amendments significantly enhances crop growth and productivity, highlighting their importance for sustainable farming practices (Salman et al., 2023). Notably, PGPR play a critical role in enhancing nutrient availability by solubilizing phosphorus, fixing atmospheric nitrogen, and producing growth-promoting hormones, thus improving root development and nutrient uptake in crops like berseem and cowpea (Sharma et al., 2020; Mahmud et al., 2021; Shah et al., 2021; Ullah et al., 2023). Additionally, Panchagavya, a traditional formulation composed of five cow-derived products - milk, curd, ghee, urine, and dung serves as a powerful bio-stimulant (Kumar et al., 2023). It promotes plant growth, enhances microbial activity in the soil, and boosts plant immunity (Golakiya et al., 2019).

We hypothesize that that the integrated application of FYM, PGPR, and Panchagavya can simultaneously enhance forage yield and quality in a multi-crop system comprising maize, berseem, and cowpea. The objective of this research is to develop and assess the effect of integrated organic nutrient management strategy aimed at producing high-quality organic fodder crops. By employing a combination of FYM, PGPR, and Panchagavya, this study seeks to enhance crop yields and improve forage quality. This research work aims to support sustainable agricultural practices, improve livestock productivity, and contribute to environmental conservation.

## Material and methods

### Experimental details

The investigation transpired at the experimental research farm situated in the Agronomy Section of the ICAR-National Dairy Research Institute, Karnal, Haryana, during the rainy, winter and summer season from 2018-2021. The soil of the experimental field (0-15 cm) was clay loam in texture with EC (0.23 dS m^-1^), pH (7.52), medium in SOC (0.601%) and medium in available potassium (190.2 kg ha^-1^), low in available N (188.4 kg ha^-1^) and high in available phosphorus (28.54 kg ha^-1^) with SOM content (1.03 ± 0.01%). Before sowing the crop, pre-sowing irrigation was applied to bring the soil to its optimum moisture level. Subsequently, the land was cross-ploughed using a tractor-drawn disc harrow, followed by rotavator and planking to achieve a fine tilth suitable for seedbed preparation. The experiment was conducted on a fixed plot with a gross plot size of 8 meters in length and 6 meters in breadth (**Supplementary Figure 1**).

The field experiment consisted of maize (M), Berseem (B) and Cowpea (C) cropping system was laid down in randomized complete block design (RCBD) with three replication viz., T_1_: 100% RDN through FYM (M) - No application (B) - No application (C); T_2_: 50% RDN through FYM + PGPR + 3% foliar spray of panchagavya (M) – PGPR + 3% foliar spray of panchagavya (B) – PGPR + 3% foliar spray of panchagavya (C); T_3_: 75% RDN through FYM + PGPR (M) - PGPR (B)- PGPR (C); T_4_: 100% RDN through FYM + PGPR (M) - PGPR (B) - PGPR (C); T_5_: 75% RDN through FYM + PGPR + 3% foliar spray of panchagavya (M) – PGPR + 3% foliar spray of panchagavya (B) – PGPR + 3% foliar spray of panchagavya (C); and T_6_: 100% RDN through FYM + PGPR + 3% foliar spray of panchagavya (M) – PGPR + 3% foliar spray of panchagavya (B) – PGPR + 3% foliar spray of panchagavya (C); T_7_: 100% RDF (M)- 100% RDF (B)- 100% RDF (C). In treatment T_7_, 100 kg N: 60 kg P_2_O_5_: 40 kg K_2_O, 20 kg N:60 kg P_2_O_5_:40 kg K_2_O and 20 kg N:60 kg P_2_O_5_ were applied as recommended fertilizer doses for maize, berseem, and cowpea crops, respectively. The N in the control treatment was applied in two splits (50 kg N) at basal and after 30 days of sowing (DAS) in maize, while in berseem and cowpea, N was applied as basal dose only. Phosphorus (P) and potassium (K) were applied as basal doses for maize, berseem and cowpea crops. A PGPR formulation “NPK liquid biofertilizer” was obtained from Division of Microbiology, ICAR-Indian Agricultural Research Institute, New Delhi for seed inoculation in present study. This formulation is consisting of three different microbial strains namely *Azotobacter chroococcum* (N_2_ fixing bacteria), *Pseudomonas straita* (P-solubilizing bacteria), and *Bacillus decolorationis* (K-solubilizing bacteria). Each bacterial strain contains 10^9^ or greater CFU mL^-1^ in formulation. FYM was applied as basal dose at the time of sowing in treatments T_1_ to T_6_ based on the N content as per the schedule, and seed treatment with PGPR solution was performed as per treatment details. The foliar spray of panchagavya was applied @ 3% (liquid formulation) at 30, 40, and 50 DAS. The mean concentration values of oxidizable organic carbon (%), total carbon (%), total N (%), total P (%), and total K (%) in FYM applied were 11.49, 21.37, 0.68, 0.45, and 0.90 respectively during three years of experimentation. The mean total N, P, and K nutrient concentration values of panchagavya applied as foliar spray were 0.65%, 0.10%, and 0.47%, respectively. For the fodder crops maize, berseem, and cowpea, the varieties J-1006, Mascavi, and C-152 were used in the experiment. These were sown at seed rates of 40 kg ha-¹, 25 kg ha-¹, and 40 kg ha-¹, respectively. The single-cut maize variety J-1006 was sown during the rainy season, the multicut berseem variety ‘Mascavi’ was sown in the winter season, and the single-cut cowpea variety C-152 was sown in the summer season. The spacing adopted for maize was 60 cm x 20 cm, while berseem was broadcast, and cowpea was planted at a spacing of 30 cm x 10 cm. A single hand weeding was conducted in the maize crop 20 days after sowing, while no weeding was performed in berseem and cowpea. To control insect pests in the fodder maize, Azadirachtin (1500 ppm) was applied at a concentration of 4 mL L^-1^ of water. No insect damage was observed in the berseem and cowpea crops.

### Preparation and characterization of panchagavya

Panchagavya was prepared using five cow-derived products. The process involved combining 15 liters of cow urine, 10 kilograms of cow dung, 4 liters of cow milk, 4 kilograms of cow curd, and 1 kilogram of cow ghee in a plastic container. To this mixture, 2 dozen ripe bananas and 2 kilograms of jaggery, both chopped into small pieces, were added. Additionally, 500 grams of turmeric powder and 1 liter of coconut water (from 4 tender coconuts) were incorporated.

The mixture was thoroughly stirred and sealed in the plastic drum. It was agitated twice daily for 25 days to ensure proper fermentation. Once the preparation was complete, the solution was filtered using a white muslin cloth, with 8 liters of distilled water added during the process. This yielded approximately 18 liters of Panchagavya, which was stored in a dark, room-temperature environment. The composition of the prepared panchagavya includes total N (0.64%), total P (0.10%), total K (0.47%), zinc (1.05 mg kg^-1^), and iron (8.75 mg kg^-1^).

### Yield and proximate composition estimation

The crops fodder maize, berseem, and cowpea were manually harvested, and their fresh weights were recorded. These yields (measured in kg per plot) were then converted to tons per hectare. Representative samples from each plot were collected systematically and dried in a hot air oven at 60°C for 48 hours. After drying, the dry fodder yield was calculated in tons per hectare. Once desiccated, the samples were ground using a Wiley mill and passed through a 1 mm sieve, then stored in sealed polyethylene bags for further analysis. The analysis of the fodder’s proximate composition, encompassing crude protein (CP), ether extract (EE), and total ash (TA), was conducted utilizing the AOAC (2005) method. The CP content was calculated by multiplying the nitrogen content measured via the Kjeldahl method by a factor of 6.25.

### Determination of fiber fractions of different crops

The methods used to determine neutral detergent fiber (NDF), acid detergent fiber (ADF), and acid detergent lignin (ADL) followed the procedures described by Van Soest et al. (1991). Acid-insoluble ash (AIA) was measured from the acid detergent fiber following the procedure outlined by Oke (2014). Neutral detergent-insoluble nitrogen (NDIN) and acid detergent-insoluble nitrogen (ADIN) were determined by analyzing the residues from NDF and ADF, respectively, using the Kjeldahl nitrogen estimation technique, as specified by Licitra et al. (1996). The values of neutral detergent insoluble crude protein (NDICP) and acid detergent insoluble crude protein (ADICP), as percentages of dry matter (DM), were calculated by taking the NDIN and ADIN values and multiplying them by 6.25. To express NDICP and ADICP as percentages of crude protein, the values, initially determined as percentages of dry matter, were divided by the sample’s CP content.

### Statistical analysis

The data collected from the field experiment was statistically analyzed using an analysis of variance (ANOVA) test to examine and compare mean values, adhering to the approach described by Gomez and Gomez (1984). LSD values (*p* ≤ 0.05) were applied to assess significant differences between treatment means. Graphical representations were created using GraphPad PRISM (version 8.0), and correlation analysis was performed with PAST software (version 4.11).

## Results

### Green and dry fodder yield in the fodder maize-berseem-cowpea cropping system

#### Fodder maize

**Figure 1:**
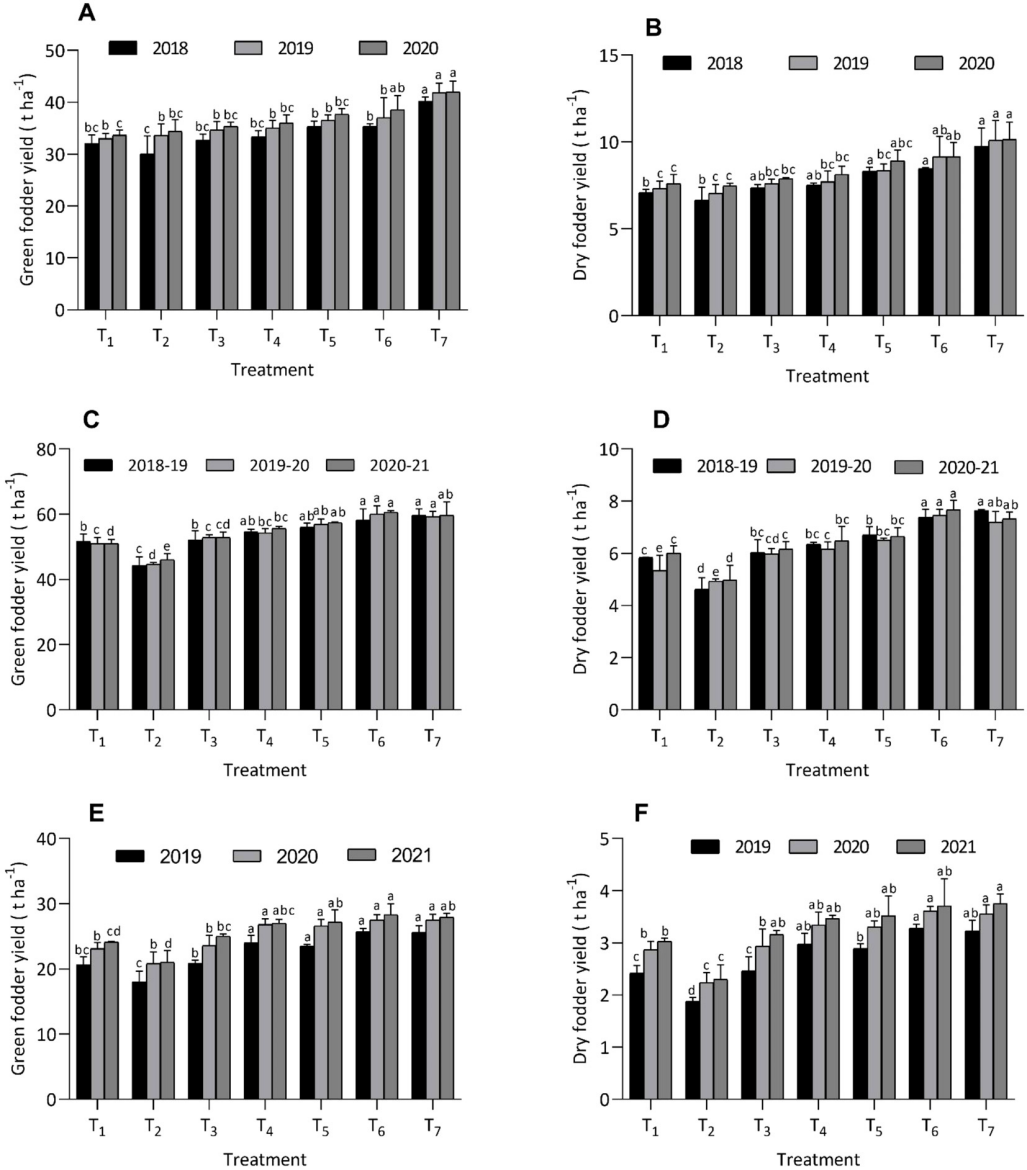
Effect of organic and inorganic nutrient amendments on yields of multi crop system: maize green fodder yield **(A)**, maize dry fodder yield **(B)**, berseem green fodder yield **(C)**, berseem dry fodder yield **(D)**, cowpea green fodder yield **(E)**, and cowpea dry fodder yield **(F)**. The data represents the mean values across treatments (T_1_-T_7_) with error bars indicating standard deviation. Statistical significance was assessed using one way ANOVA, with differences considered significant at *p* ≤ 0.05. Different lowercase letters indicate significant differences among different treatments based on LSD test.

The study evaluated the green and dry fodder yield of fodder maize under different integrated organic nutrient amendments over three years. Treatment T_6_ consistently achieved the highest green fodder yield across all years, with values of 35.4 ± 0.5, 37.0 ± 3.9, and 38.6 ± 2.7 t ha^-1^, demonstrating parity with treatments T_1_, T_3_, T_4_, and T_5_ but outperforming T_2_ in 2018. In 2019, T_6_ showed parity with all organic treatments, while in 2020, it was on par with T_2_, T_3_, T_4_, and T_5_. The lowest green fodder yields were recorded under T_2_ in 2018 and T_1_ in 2019 and 2020. Regarding dry fodder yield, T_6_ again led across all years, showing parity with T_3_, T_4_, and T_5_ but exceeding T_2_. Treatment T_7_, with inorganic fertilizers, produced the highest green and dry fodder yields in 2018, performing similarly to T_6_ in 2019 and 2020 (**Figures 1A, B**).

#### Berseem

The study assessed the impact of various integrated organic nutrient amendments on the green and dry fodder yield of berseem over three years. Among the organic treatments, treatment T_6_ consistently recorded the highest green fodder yields, with values of 58.2 ± 3.41 t ha^-1^ in 2018-19, 60.0 ± 2.58 t ha^-1^ in 2019-20, and 60.6 ± 0.50 t ha^-1^ in 2020-21. These yields were statistically on par with treatments T_7_ and T_5_. Conversely, treatment T_2_ produced the lowest green fodder yields across all three years. Regarding dry fodder yield, treatment T_6_ again led, with yields of 7.39 ± 0.30 t ha^-1^ in 2018-19, 7.45 ± 0.24 t ha^-1^ in 2019-20, and 7.67 ± 0.37 t ha^-1^ in 2020-21, while treatment T_2_ recorded the lowest yields (**Figures 1C, D**). The findings highlight the superior performance of treatment T_6_ in both green and dry fodder yields compared to other organic amendments.

#### Cowpea

The study observed significant variation in the green fodder yield of cowpea under different integrated organic nutrient amendments. Treatment T_6_ consistently produced the highest green fodder yield across three years (25.7 ± 0.51, 27.5 ± 0.80, and 28.3 ± 1.67 t ha^-1^), which was statistically comparable to treatments T_4_, T_5_, and T_7_. Notably, T_6_ significantly outperformed treatments T_1_, T_2_, and T_3_ in all three years. Conversely, the lowest green fodder yields were recorded in treatment T_3_ (18.0 ± 1.68, 20.8 ± 1.79, and 21.0 ± 1.83 t ha^-1^). Additionally, T_6_ achieved the highest dry fodder yield (3.28 ± 0.08, 3.61 ± 0.09, and 3.71 ± 0.52 t ha^-1^), showing a significant advantage over treatment T_2_, which had the lowest dry fodder yield (1.88 ± 0.07, 2.24 ± 0.19, and 2.30 ± 0.28 t ha^-1^) during the 2019, 2020 and 2021, respectively (**Figures 1E, F**).

#### Proximate composition

**Figure 2:**
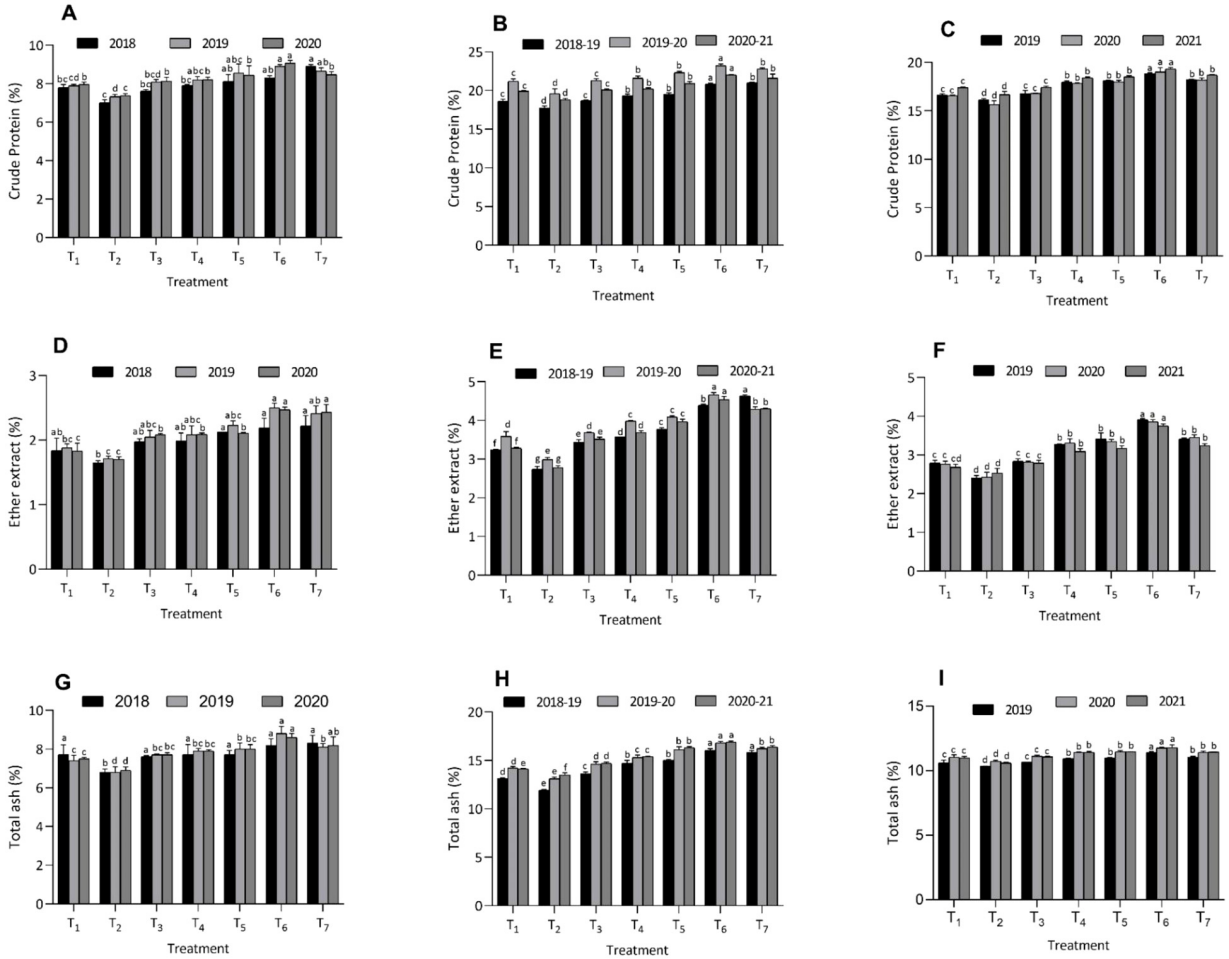
Effect of organic and inorganic nutrient amendments on proximate composition of multi crop system: maize crude protein content **(A)**, berseem crude protein content **(B)**, cowpea crude protein content **(C)**, maize ether extract content **(D)**, berseem ether extract content **(E)**, cowpea ether extract content **(F)**, maize total ash content **(G)**, berseem total ash content **(H)**, and cowpea total ash content **(I)**. The data represents the mean values across treatments (T_1_-T_7_) with error bars indicating standard deviation. Statistical significance was assessed using one way ANOVA, with differences considered significant at *p* ≤ 0.05. Different lowercase letters indicate significant differences among different treatments based on LSD test.

The application of integrated organic nutrient amendments significantly impacted the proximate composition of various crops, specifically influencing CP, EE, and TA contents (**Figures 2A–I**). In maize, treatment T_6_ demonstrated superior results with the highest CP (8.30 ± 0.11% in 2018, 10.1 ± 0.09% in 2019, and 10.2 ± 0.13% in 2020), EE (2.19 ± 0.15% in 2018, 2.50 ± 0.07% in 2019, and 2.47 ± 0.04% in 2020), and TA content (8.20 ± 0.33% in 2018, 8.80 ± 0.36% in 2019, and 8.60 ± 0.20% in 2020). Treatment T_6_ found at par with T_1_, T_3_, T_5_, and T_7_ for CP, EE and TA content in berseem crop during all the three years. In contrast, treatment T_2_ showed the lowest values in all measured parameters across the same years. Similarly, in berseem, T_6_ consistently achieved the highest levels of CP (20.8 ± 0.14% in 2018-19, 23.2 ± 0.22% in 2019-20, and 22.0 ± 0.07% in 2020-21), EE (4.39 ± 0.03% in 2018-19, 4.66 ± 0.06% in 2019-20, and 4.54 ± 0.08% in 2020-21), and TA content (16.0 ± 0.17% in 2018-19, 16.8 ± 0.16% in 2019-20, and 16.9 ± 0.09% in 2020-21), compared to the lower values recorded by T_2_.

In cowpea, treatment T_6_ also yielded significantly higher CP (18.9 ± 0.08% in 2019, 19.0 ± 0.42% in 2020, and 19.3 ± 0.13% in 2021), EE (3.91 ± 0.03% in 2019, 3.86 ± 0.05% in 2020, and 3.75 ± 0.05% in 2021), and TA content (11.4 ± 0.09% in 2019, 11.8 ± 0.08% in 2020, and 11.8 ± 0.22% in 2021). Treatment T_6_ recorded significant superiority over T_1_, T_2_, T_3_, T_4_, T_5_, and T_7_ for CP, EE and TA content in cowpea crop during all the three years. Again, T_2_ was associated with the lowest values for these parameters throughout the respective years. Overall, the findings highlight the superior efficacy of treatment T_6_ in enhancing the nutritional quality of maize, berseem, and cowpea crops through integrated organic nutrient amendments, suggesting its potential for improving crop quality in sustainable agricultural practices.

#### Fiber fractions

**Figure 3:**
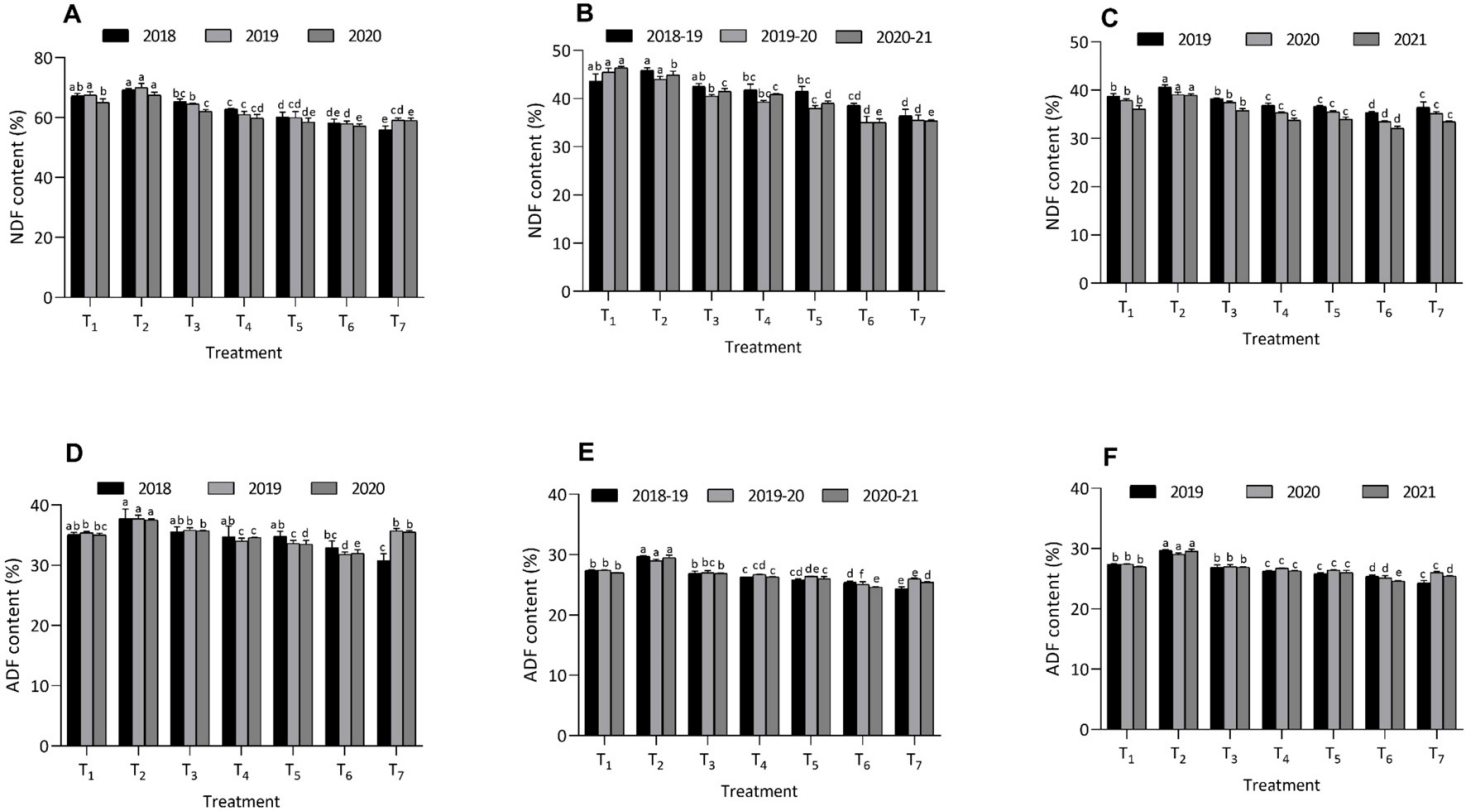
Effect of organic and inorganic nutrient amendments on fiber fractions of multi crop system: maize NDF content **(A)**, berseem NDF content **(B)**, cowpea NDF content **(C)**, maize ADF content **(D)**, berseem ADF content **(E)**, and cowpea ADF content **(F)**. The data represents the mean values across treatments (T_1_-T_7_) with error bars indicating standard deviation. Statistical significance was assessed using one way ANOVA, with differences considered significant at *p* ≤ 0.05. Different lowercase letters indicate significant differences among different treatments based on LSD test.

**Figure 4:**
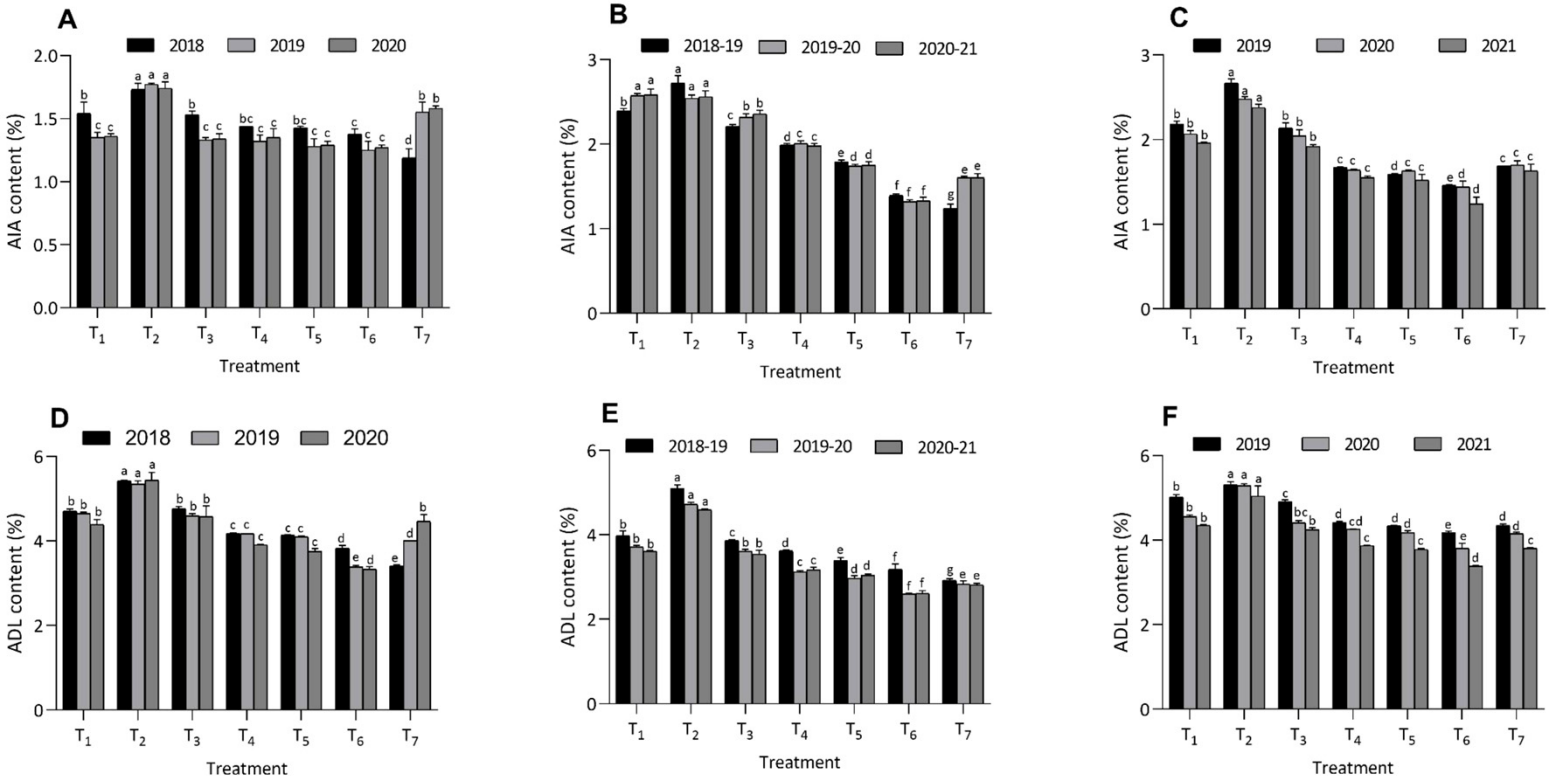
Effect of organic and inorganic nutrient amendments on fiber fractions of multi crop system: maize ADL content **(A)**, berseem ADL content **(B)**, cowpea ADL content **(C)**, maize AIA content **(D)**, berseem AIA content **(E)**, and cowpea AIA content **(F)**. The data represents the mean values across treatments (T_1_-T_7_) with error bars indicating standard deviation. Statistical significance was assessed using one way ANOVA, with differences considered significant at *p* ≤ 0.05. Different lowercase letters indicate significant differences among different treatments based on LSD test.

The study explored the influence of integrated organic nutrient amendments on the fiber content of fodder crops, focusing on maize, berseem, and cowpea (**Figures 3A–F** and **Figures 4A–F**). The fiber fractions measured included NDF, ADF, ADL, and AIA. Among the treatments, treatment T_6_ consistently resulted in the most significant reduction in these fiber fractions across all three crops. In fodder maize, treatment T_6_ significantly lowered the levels of NDF (58.2 ± 1.31%, 58.0 ± 0.90%, 57.2 ± 0.68%), ADF (32.9 ± 1.16%, 31.8 ± 0.42%, 32.0 ± 0.57%), ADL (3.83 ± 0.06%, 3.39 ± 0.03%, 3.33 ± 0.06%), and AIA (1.38 ± 0.04%, 1.25 ± 0.07%, 1.27 ± 0.02%) compared to the other treatments. Treatment T_7_ recorded the lowest values of NDF, ADF, ADL and AIA during 2018 compared to T_6_. During 2019 and 2020, T_6_ recorded significantly lower values of NDF, ADF, ADL and AIA content compared to remaining treatments. The highest fiber fractions were observed in treatment T_2_, where NDF, ADF, ADL, and AIA were recorded at significantly higher values during all the three years.

Similarly, in berseem and cowpea, treatment T_6_ also led to the lowest fiber fractions, with NDF, ADF, ADL, and AIA levels substantially reduced compared to the other treatments. In berseem, T_6_ recorded NDF at 38.6 ± 0.4%, 35.0 ± 1.30%, 35.0 ± 0.79%, ADF at 25.4 ± 0.23%, 25.1 ± 0.40%, 24.6 ± 0.09%, ADL at 3.18 ± 0.13%, 2.60 ± 0.02%, 2.61 ± 0.07%, and AIA at 1.39 ± 0.02%, 1.32 ± 0.02%, 1.33 ± 0.04%. Treatment T_7_ recorded the lowest values of NDF, ADF, ADL and AIA during 2018-19 compared to T_6_. During 2019-20 and 2020-21, T_6_ recorded significantly lower values of NDF, ADF, ADL and AIA content compared to remaining treatments. In cowpea, treatment T_6_ produced NDF values of 35.4 ± 0.22%, 33.4 ± 0.25%, 32.1 ± 0.42%, ADF at 27.4 ± 0.16%, 26.6 ± 0.30%, 27.2 ± 0.06%, ADL at 4.18 ± 0.03%, 3.80 ± 0.12%, 3.38 ± 0.03%, and AIA at 1.46 ± 0.01%, 1.44 ± 0.07%, 1.24 ± 0.08%. Treatment T_7_ recorded the lowest values of NDF, ADF, ADL and AIA during 2018-19 compared to T_6_. During 2019-20 and 2020-21, T_6_ recorded significantly lower values of NDF, ADF, ADL and AIA content compared to remaining treatments. The highest fiber fractions for these crops were recorded under treatment T_2_, indicating its lesser effectiveness. These findings suggest that the use of integrated organic nutrient amendments, particularly T_6_, is highly effective in improving the nutritional quality of fodder crops by reducing their fiber content.

**Figure 5:**
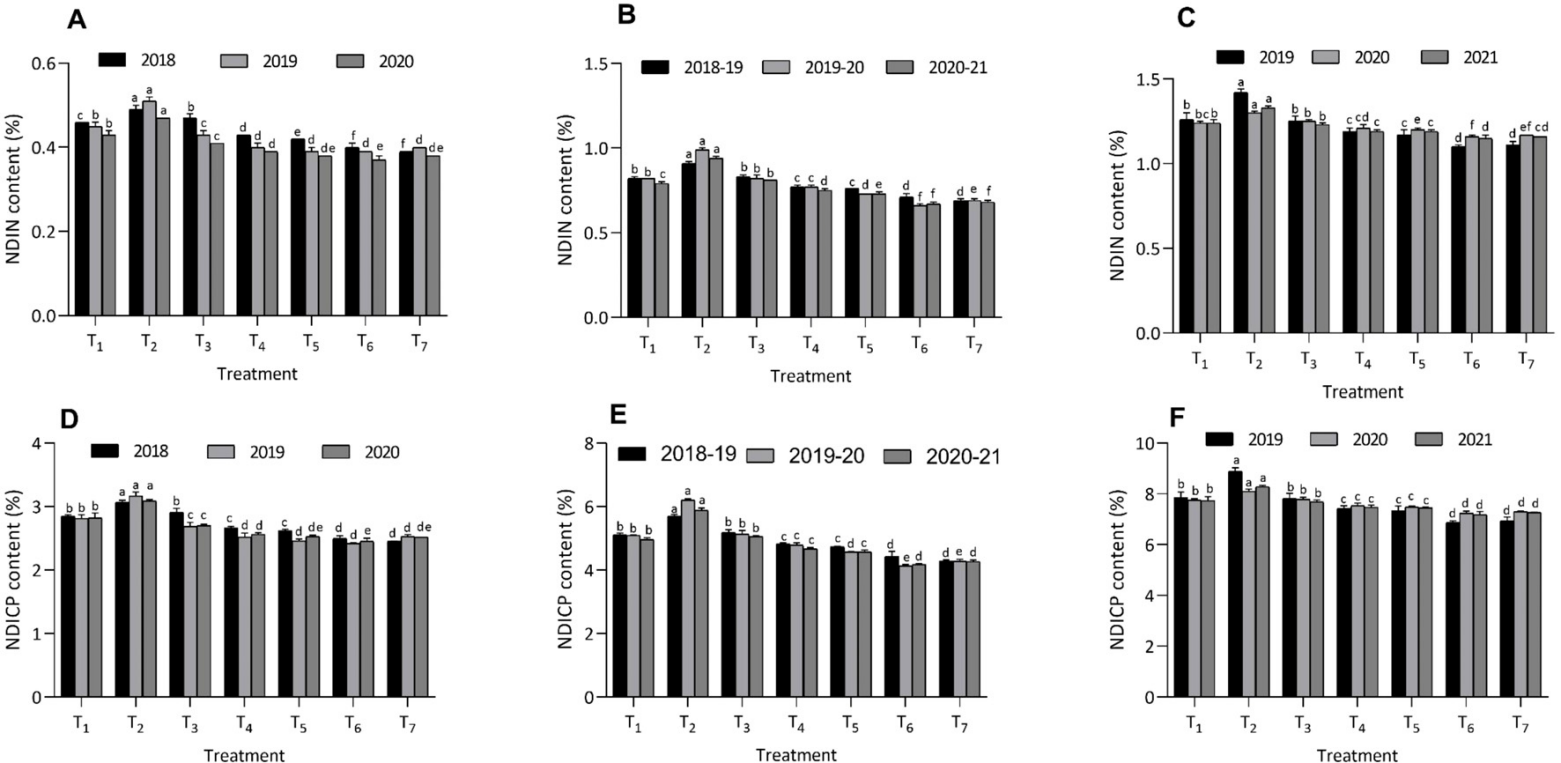
Effect of organic and inorganic nutrient amendments on fiber fractions of multi crop system: maize NDIN content **(A)**, berseem NDIN content **(B)**, cowpea NDIN content **(C)**, maize NDICP content **(D)**, berseem NDICP content **(E)**, and cowpea NDICP content **(F)**. The data represents the mean values across treatments (T_1_-T_7_) with error bars indicating standard deviation. Statistical significance was assessed using one way ANOVA, with differences considered significant at *p* ≤ 0.05. Different lowercase letters indicate significant differences among different treatments based on LSD test.

**Figure 6:**
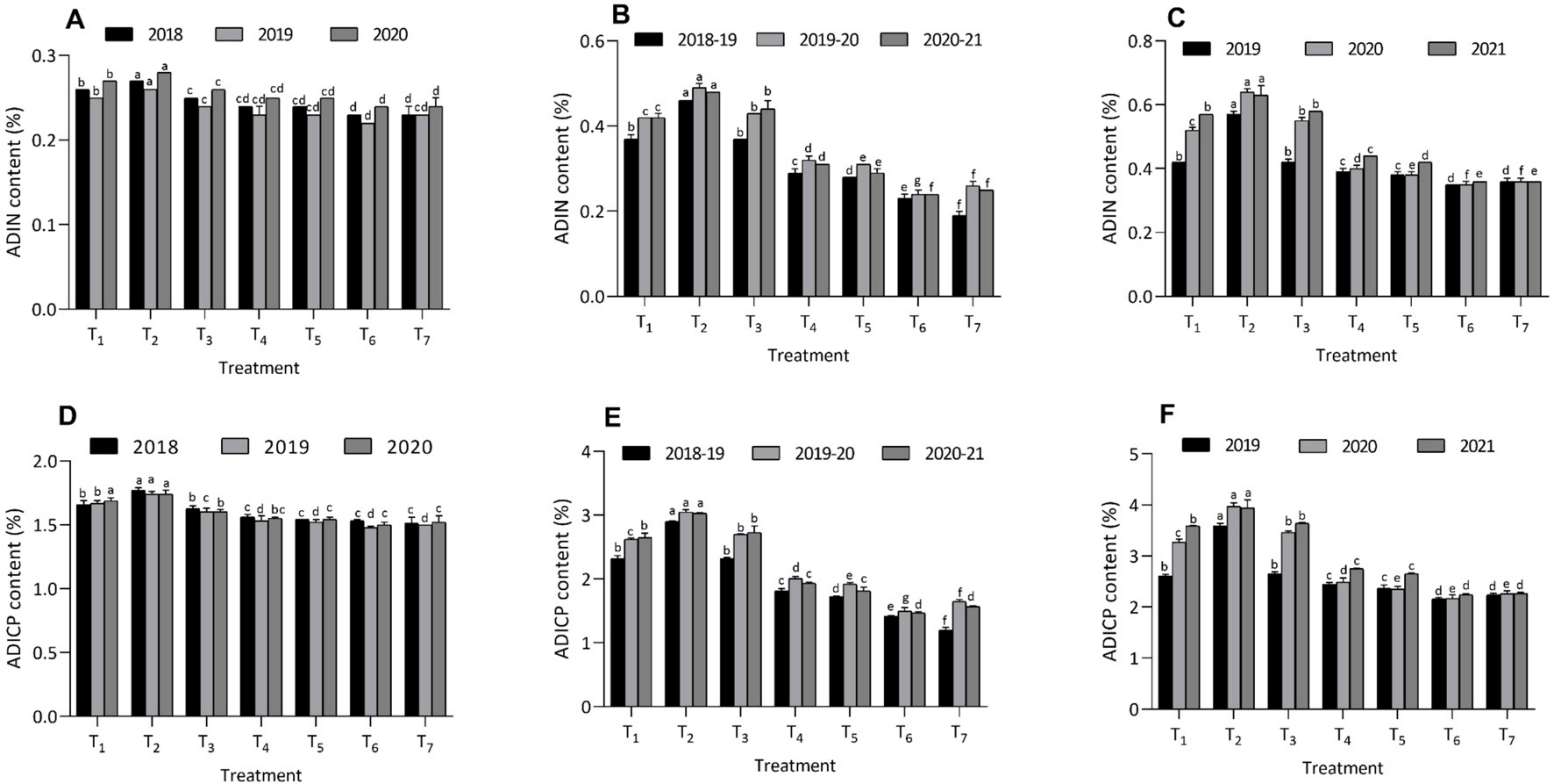
Effect of organic and inorganic nutrient amendments on fiber fractions of multi crop system: maize ADIN content **(A)**, berseem ADIN content **(B)**, cowpea ADIN content **(C)**, maize ADICP content **(D)**, berseem ADICP content **(E)**, and cowpea ADICP content **(F)**. The data represents the mean values across treatments (T_1_-T_7_) with error bars indicating standard deviation. Statistical significance was assessed using one way ANOVA, with differences considered significant at *p* ≤ 0.05. Different lowercase letters indicate significant differences among different treatments based on LSD test.

Across various organic amendments, the lowest NDIN values were observed in the T_6_ treatment, recording 0.40 ± 0.01, 0.39 ± 0.00, and 0.37 ± 0.01 in maize; 0.71 ± 0.02, 0.66 ± 0.01, and 0.67 ± 0.01 in berseem; and 1.10 ± 0.01, 1.16 ± 0.01, and 1.15 ± 0.00 in cowpea over three years. The highest NDIN values were recorded under the T_2_ treatment, with 0.49 ± 0.01, 0.51 ± 0.01, and 0.47 ± 0.00 in maize; 0.91 ± 0.01, 0.99 ± 0.01, and 0.94 ± 0.01 in berseem; and 1.42 ± 0.02, 1.30 ± 0.01, and 1.33 ± 0.01 in cowpea during the same period. Additionally, the lowest NDICP values were observed in the T_6_ treatment, with values of 2.50 ± 0.04, 2.42 ± 0.01, and 2.45 ± 0.05 in maize; 4.43 ± 0.16, 4.14 ± 0.04, and 4.17 ± 0.04 in berseem; and 6.87 ± 0.07, 7.23 ± 0.09, and 7.19 ± 0.11 in cowpea on a dry matter basis over three years. In contrast, the highest NDICP values were found under the T_2_ treatment, with 3.07 ± 0.03, 3.17 ± 0.06, and 3.09 ± 0.02 in maize; 5.70 ± 0.05, 6.20 ± 0.04, and 5.88 ± 0.08 in berseem; and 8.89 ± 0.14, 8.10 ± 0.09, and 8.28 ± 0.06 in cowpea across the same period (**Figures 5A-F**). The values of NDIN and NDICP in treatment T_6_ were statistically comparable to those in the T_7_ treatment. Similar patterns were also observed in the ADIN and ADICP values for all three crops over the three-year study period (**Figures 6A–F**).

### Impact of organic amendments on yield, proximate composition, and fiber fractions in different crops

**Figure 7:**
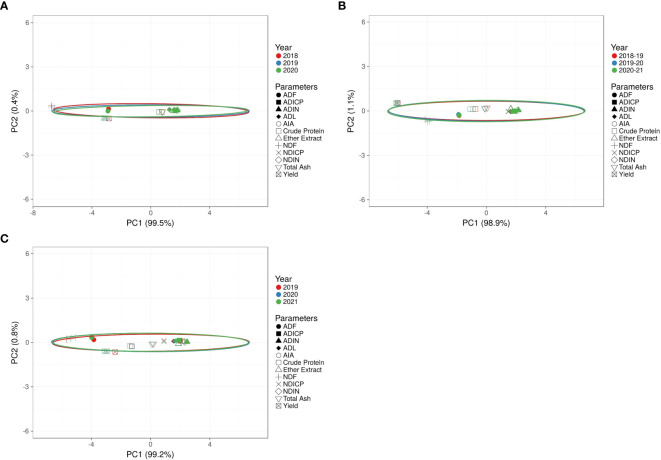
**(A)** Effect of organic and inorganic nutrient amendments on principal component analysis of maize in multi crop system. **(B)** Effect of organic and inorganic nutrient amendments on principal component analysis of berseem in multi crop system. **(C)** Effect of organic and inorganic nutrient amendments on principal component analysis of cowpea in multi crop system.

The evaluation of organic amendments, including PGPR, FYM, and foliar application of panchagavya, on crop yield, proximate composition, and fiber fractions was conducted using principal component analysis (PCA) and correlation matrix analysis. The PCA biplot for maize (**Figure 7A**) showed that PC1 accounted for 99.5% of the variance, while PC2 accounted for 0.4%. The ellipses representing different years overlapped significantly, indicating minimal inter-annual variation. Parameters such as ADF, ADICP, NDICP, and NDF were closely clustered, reflecting their similar contributions to the variance. Most data points were tightly grouped around the origin, suggesting limited variability across treatments. For berseem (**Figure 7B**), PC1 explained 98.9% of the variance, and PC2 explained 1.1%. The ellipses for different years overlapped considerably, with ADF, ADICP, NDICP, and NDF closely associated, contributing similarly to the variance. The concentration of data points near the origin indicated minimal parameter spread across years. In cowpea (**Figure 7C**), PC1 accounted for 99.2% of the variance, while PC2 accounted for 0.8%. Similar to maize and berseem, the ellipses for each year overlapped significantly, showing consistent performance across years. Parameters such as ADF, ADICP, NDICP, and NDF were tightly clustered, indicating comparable contributions to the overall variance. The majority of data points were concentrated near the origin, demonstrating limited parameter variability across treatments.

The correlation matrices from 2018, 2019, and 2020 showed relationships between maize yield, CP, EE, TA, and fiber components like NDF, ADF, and ADL (**Supplementary Figure 2A, C**). Yield consistently exhibits a positive correlation with CP and EE content, but negative correlations with fiber components (NDF, ADF, ADL, NDIN, and ADIN). This suggests that higher yield is linked to increased protein and fat content but lower fiber content. The CP content shows strong positive correlations with EE and negative correlations with fiber components, indicating that maize with higher protein content tends to have lower fiber. The EE content mirrors this pattern, being positively related to CP content and negatively correlated with fiber fractions. Fiber components, including NDF, ADF, and ADL, are strongly correlated with each other and negatively correlated with yield, CP, and EE, highlighting a trade-off between high fiber content and other desirable traits.

Further, the correlation matrix reveals notable relationships between yield, CP, fiber, and other components of berseem and cowpea (**Supplementary Figures 3A–C** and **Supplementary Figures 4A–C**). Yield is negatively correlated with NDF, ADF, and ADL, indicating that increased fiber content tends to reduce yield, while it shows positive correlations with CP and EE content, suggesting that higher CP and EE levels are associated with greater yields. The CP content exhibits a strong positive correlation with EE, implying that higher CP content is often accompanied by higher EE levels. In contrast, CP content is negatively correlated with fiber components like NDF, ADF, and ADL. The fiber variables are strongly positively correlated with each other, showing that when one fiber component increases, others tend to follow. The TA content displays negative correlations with NDF and ADF but positive correlations with yield, CP, and EE content. These relationships highlight the inverse association between fiber content and both yield and CP content.

## Discussion

The findings of our study emphasize the significant influence of integrated organic nutrient amendments on green and dry fodder yields in a fodder maize-berseem-cowpea cropping system. In maize, T_6_ exhibited superior green and dry fodder yields compared to the other treatments, particularly surpassing T_2_, which underscores the advantage of utilizing comprehensive organic amendments over lower input alternatives. The superior performance of T_6_ in green and dry fodder yields can be attributed to the combined effects of FYM, PGPR, and panchagavya, which enhance nutrient availability, uptake, and overall plant growth. Kumar et al. (2021b) reported that application of farmyard manure improves the soil biological activity, increase the nutrient availability leading to increase in maize yield under sub-tropical climatic conditions. Further, Moridi et al. (2019) observed that in the calcareous soils of Iran, PGPR improve nutrient availability by aiding in the decomposition of organic matter in farmyard manure, which releases nutrients and supports plant growth. Similar findings were reported by (Choudhary et al., 2024a) and (Shilpa Sharma et al, 2024) in sub-tropical climatic conditions. Savaliya et al. (2023) reported that foliar application of panchagavya enhances the yield of cowpea under tropical climatic conditions. Kumar et al. (2023) reported that combined application of panchagavya with organic sources of nutrient leads to increase in crop yield. Treatment T_7_, which received 100% RDF, achieved the highest fodder yields due to immediate nutrient supply from inorganic fertilizers. Similar results were reported by Otieno et al. (2021) for maize crop in the tropical climate of Kenya. The lowest yields in T_2_ can be attributed to its insufficient nitrogen input and fewer organic amendments, limiting nutrient availability and plant growth potential. The lower yields in treatment T_1_ compared to T_6_ and T_7_ may be attributed to the slower nutrient release from FYM in the maize crop during the rainy season, coupled with the absence of subsequent nutrient application in the berseem and cowpea crops during the winter and summer seasons, respectively. Mahmood et al. (2017) reported that application of organic manure results into lower yield due to slow decomposition of organic matter compared to inorganic fertilizers in maize crop under semi-arid climatic conditions. Further, treatment T_3_, T_4_ and T_5_ recorded lower yield in comparison to T_6_ and T_7_ may be attributed to slow nutrient release from FYM decomposition and lower nutrient application in different crops in different seasons. The current findings resonate with those of Mahmood et al. (2017) and (Sigaye et al., 2020).

The results of this study demonstrate the substantial impact of integrated organic nutrient amendments on the proximate composition of fodder maize-berseem-cowpea cropping system, specifically in terms of CP, EE, and TA content. Among the organic amendments, T_6_, consistently exhibited superior quality across all three crops over the three-year period. T_6_ significantly enhanced CP, EE, and TA content. This rise in proximate composition under T_6_ may be attributed to integrated organic nutrient amendments that provides a balanced nutrient supply, promotes better nutrient uptake, and improves the overall health of the cropping system. Ngone et al. (2023) reported that of biofertilizers along with manures increased the CP, EE and TA content in maize crop in tropical climate of Cameroon. Choudhary et al. (2024b) reported that integrated application of PGPR, with organic and inorganic sources of plant nutrient results in increased CP, EE and TA content in Chinese cabbage under subtropical conditions. The application of various blended fertilizers enhances nutrient availability, supporting the growth and development of sorghum crops in the semi-arid lowland plains of Ethiopia (Weldegebriel et al., 2018). Similarly, findings were reported in *Magnolia wufengensis* by Deng et al. (2019) in temperate regions of China. The enhanced proximate composition in treatment T_7_ may be attrinuted to rapid nutrient release from inorganic fertilizers, facilitating immediate plant uptake. Anamayi et al. (2016) reported that inorganic fertilizers application recorded higher levels of CP, EE and TA content in *Moringa oleifera* in Nigeria. In contrast, T_2_ consistently exhibited the lowest values for all measured parameters, indicating that the reduced application of organic amendments resulted in nutrient deficiencies. These deficiencies limited the availability of essential minerals, fatty acids, and nitrogen, which in turn led to lower levels of CP, EE, and TA content. Nirere et al. (2021) in the tropical climatic conditions in maize crop and Yousaf et al. (2021) in radish crop under arid climatic conditions reported that low nitrogen availability results into low CP content in the crop plant. The lower CP, EE, and TA content in T_3_, T_4_, and T_5_ may be attributed to the lower nutrient application, as limited nutrient availability restricts the synthesis of proteins, lipids, and minerals, leading to decreased accumulation of these components in plant tissues. Mohan et al. (2017) reported that low nutrient availability leads to reduced synthesis of ether extract and total ash content in teosinte.

This reduction in fiber content is particularly important as lower NDF and ADF levels are associated with improved digestibility and feed quality, which are crucial factors for livestock nutrition. The study provides valuable insights into the effects of integrated organic nutrient amendments that highlight the superior performance of treatment T_6_, in reducing the fiber fractions across all three crops over the three-year study period. The significant reduction in NDF, ADF, ADL, and AIA levels observed in treatment T_6_ can be attributed to the combined application of 100% RDN through FYM, PGPR, and foliar panchagavya, which likely enhanced nutrient availability and improved nutrient uptake by the plants. This synergistic effect promotes more efficient plant metabolism, resulting in increased production of cell contents, such as proteins and sugars, while reducing the accumulation of structural carbohydrates and lignin, which contribute to higher fiber fractions. Kumar et al. (2021a) reported that integrated application of nutrient through PGPR, FYM and foliar spray of panchagavya reduced the NDF, ADF, ADL and AIA content in oats by increasing the nutrient availability. Further, in present study, treatment T_7_ recorded lower values of NDF, ADF, ADL and AIA content in all the three crops during first year compared to all the organic amendments but during second and third year of experimentation T_6_ recorded significantly lower values of NDF, ADF, ADL and AIA. This can be attributed to treatment T_7_ initially providing rapid nutrient release, promoting less fibrous tissue (Xu et al., 2020). Over time, however, prolonged use of T_7_ depletes soil organic matter, reduces microbial activity, and creates nutrient imbalances, leading to higher NDF, ADF, ADL and AIA content (Howe et al., 2024). The higher NDF, ADF, ADL, and AIA content in T_3_, T_4_, and T_5_ compared to T_6_ and T_7_ can be attributed to lower nutrient application. Insufficient nutrients hinder optimal plant growth, leading to more fibrous tissue and higher lignification (Moore et al., 2020). In contrast, treatment T_2_, which recorded the highest fiber fractions, may have provided a less efficient nutrient supply, leading to suboptimal nutrient uptake and less effective metabolic enhancement. Consequently, plants in T_2_ likely produced more cell wall material, resulting in higher NDF, ADF, ADL, and AIA levels. Similar findings were reported by Kumari et al. (2014) and Baljeet et al. (2020).

In addition to reducing fiber fractions, treatment T_6_ also consistently produced lower values of NDIN, NDICP, ADIN, and ADICP across all three crops. The lower NDIN, NDICP, ADIN, and ADICP values in treatment T_6_ across all crops can be attributed to the enhanced nutrient availability provided by organic amendments such as FYM, PGPR, and foliar panchagavya spray. These amendments likely facilitated better decomposition of organic matter and stimulated microbial activity in the soil, reducing nitrogen binding to indigestible fiber fractions like lignin and cellulose. Consequently, nitrogen became more digestible and available for plant uptake, improving its utilization by livestock and promoting nutrient cycling. These finds resonate with previous researchers Choudhary et al. (2024b) and Thomas et al. (2019). In our study, treatment T_7_ showed parity with T_6_ in terms of NDIN, NDICP, ADIN, and ADICP content in different crops, which can be attributed to the initial nutrient boost from inorganic fertilizers. This facilitated faster plant growth and improved nitrogen assimilation, reducing nitrogen binding to fiber fractions like lignin and cellulose (Talbot and Treseder, 2012). The increase in NDIN, NDICP, ADIN, and ADICP content in different crops in treatments T_1_, T_3_, T_4_, and T_5_, compared to T_6_ and T_7_, may be attributed to limited nutrient availability from lower doses of organic nutrient amendments. This resulted in slower plant growth and inefficient nitrogen assimilation, leading to more nitrogen being bound to indigestible fiber fractions like lignin and cellulose, thereby increasing NDIN, NDICP, ADIN, and ADICP content. In contrast, T_2_, with lowest organic amendment levels experienced insufficient organic matter decomposition and limited nutrient release, resulting in higher NDIN, NDICP, ADIN, and ADICP values. Lower organic inputs may have hindered microbial activity, trapping nitrogen in indigestible fiber components, thus reducing its availability and overall feed quality. Johnson et al. (2001) reported that lower application of nutrient results into increased values of NDIN, NDICP, ADIN, and ADICP in tropical grasses.

The PCA results clearly demonstrate the effectiveness of organic amendments, such as FYM, PGPR, and foliar panchagavya, in improving the consistency of crop performance across maize, berseem, and cowpea. The dominant contribution of PC1, explaining over 98% of the variance in all three crops, highlights the stability of key parameters like ADF, ADICP, NDICP, and NDF across treatments and years. The tight clustering of these fiber-related traits near the origin suggests minimal variability in nutrient composition and fiber fractions, reflecting uniform responses to the organic treatments. Additionally, the overlapping ellipses representing different years further emphasize the consistent crop performance, with little interannual variation. These findings underscore the potential of organic nutrient management strategies in maintaining stable yields and quality. Notably, the T_6_ treatment emerged as the most effective, consistently improving both yield and fiber content across all crops, supporting the sustainability of integrated organic approaches in enhancing productivity.

The findings from correlation matrix reveal a complex interaction between yield, protein, ether extract, and fiber content across maize, berseem, and cowpea crops, providing valuable insights for breeding strategies. Negative correlations between yield and fiber components (NDF, ADF, ADL) suggest that increasing yield may lead to reduced fiber content, which can enhance digestibility, particularly for livestock feed. Conversely, the positive correlations between yield, CP, and EE content indicate that crops with higher yields tend to have enhanced nutritional profiles, characterized by increased protein and ether extract content. However, the trade-off is evident, as higher fiber levels are associated with reduced protein and ether extract content, which could impact the crop’s value in bioenergy or livestock feed applications requiring high fiber. The strong association between CP and EE content implies that increasing CP also enhances EE content, thus boosting the crop’s overall nutritional quality. These insights underscore the importance of balancing yield with fiber and nutritional factors, enabling breeders to optimize crop varieties for specific agricultural and industrial needs.

## Conclusion

Integrated organic nutrient amendments are crucial for improving soil health, enhancing crop yield, and boosting the nutritional quality of crops in a sustainable, environmentally friendly way. By naturally replenishing soil nutrients, they reduce dependence on chemical fertilizers and promote more resilient agricultural systems. In this study found we found that treatment T_6_ applied with integrated amendments (FYM, PGPR and panchagavya spray) consistently outperformed other organic treatments, achieving the highest green and dry fodder yields in maize, berseem, and cowpea. T_6_ also significantly improved the nutritional profile of these crops by increasing crude protein, ether extract, and ash content, while reducing fiber fractions like NDF and ADF. Over three consecutive years, T_6_ demonstrated its superior effectiveness in enhancing both crop productivity and quality. These findings underscore the importance of integrated organic nutrient amendments in advancing sustainable agriculture, positioning T_6_ as a key strategy to boost yields, improve nutrition, and contribute to global goals for sustainable food systems and healthier ecosystems.

## Data Availability

The original contributions presented in the study are included in the article/**Supplementary Material.** Further inquiries can be directed to the corresponding author.
