# Supplementary material for: Optimizing fodder yield and quality through integrated organic nutrient amendments in multi-crop system
Source: Front Plant Sci. 2025 Feb 12;16:1517399. doi: 10.3389/fpls.2025.1517399 (PMC11860900; doi:10.3389/fpls.2025.1517399)
Supplement: Supplementary file 1 [file DataSheet1.pdf]

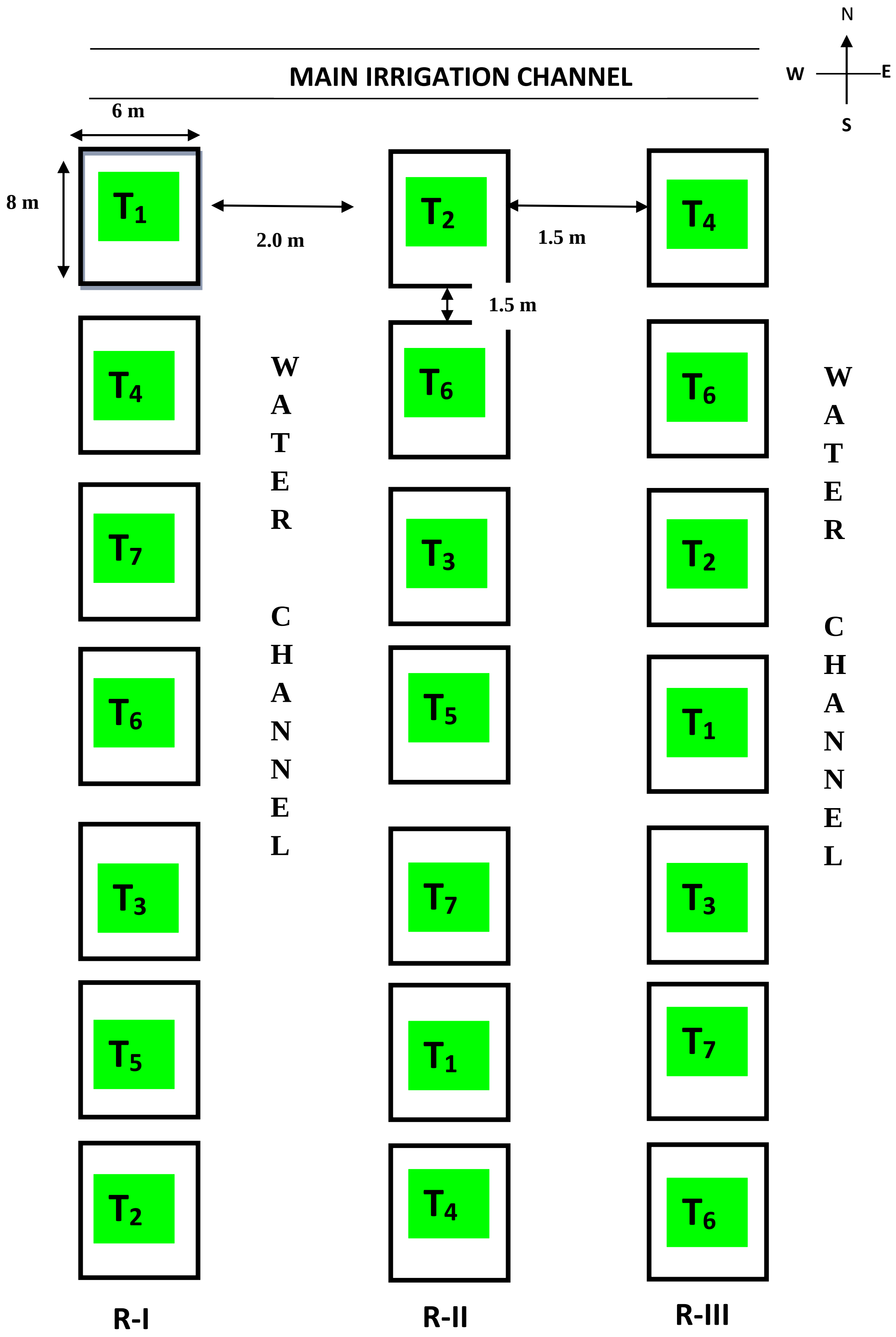

Suppl. Fig. 1. Field layout of experiment

(*R1, RII and RIII represents replication 1, 2 and 3 respectively, **Gross plot size: 8m x 6m, Net plot size: 7.5 m x 5.5 m)

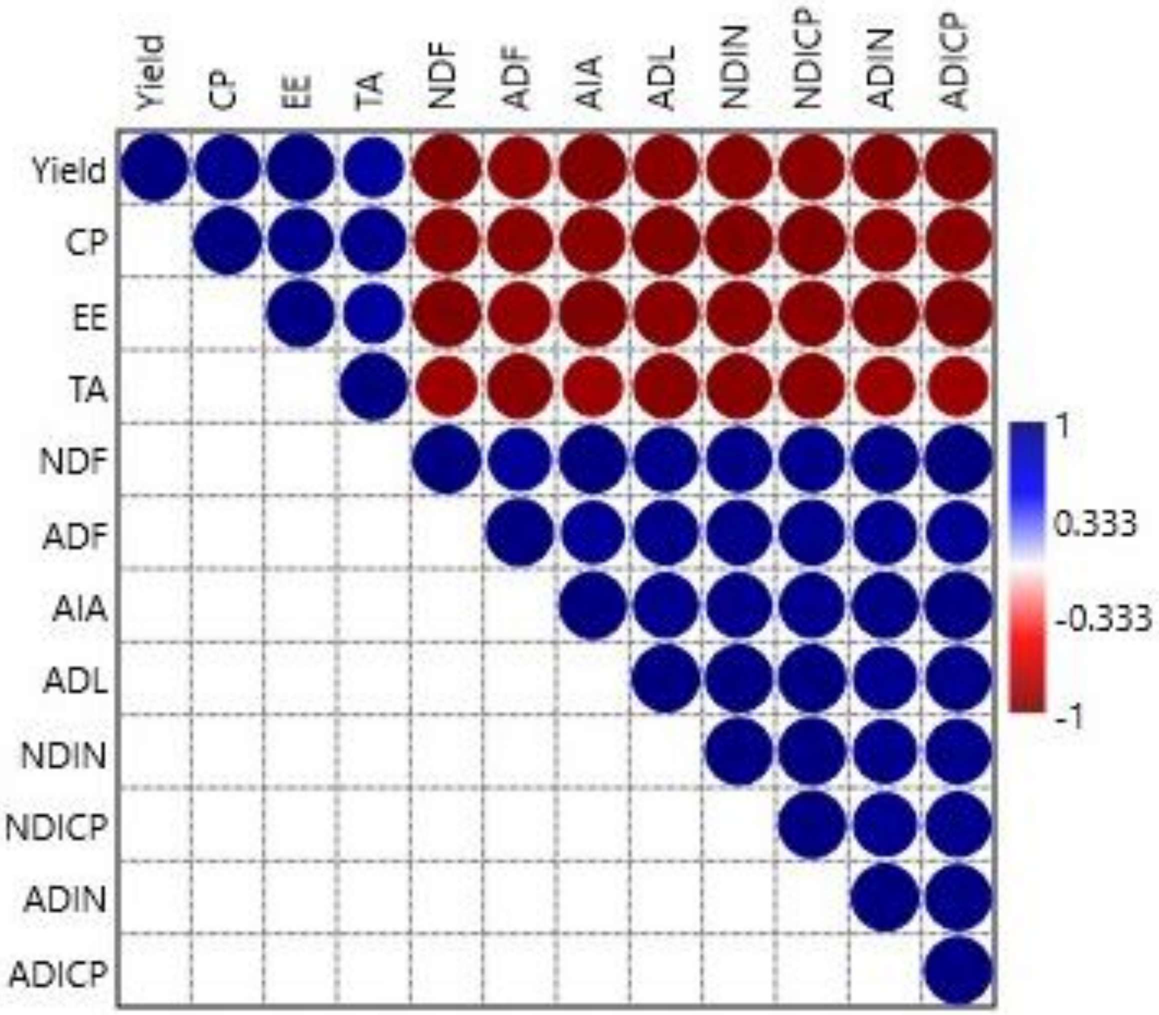

Suppl. Fig. 2A. Correlation plot matrix among yield, proximate composition and fiber fractions in maize during 2018.

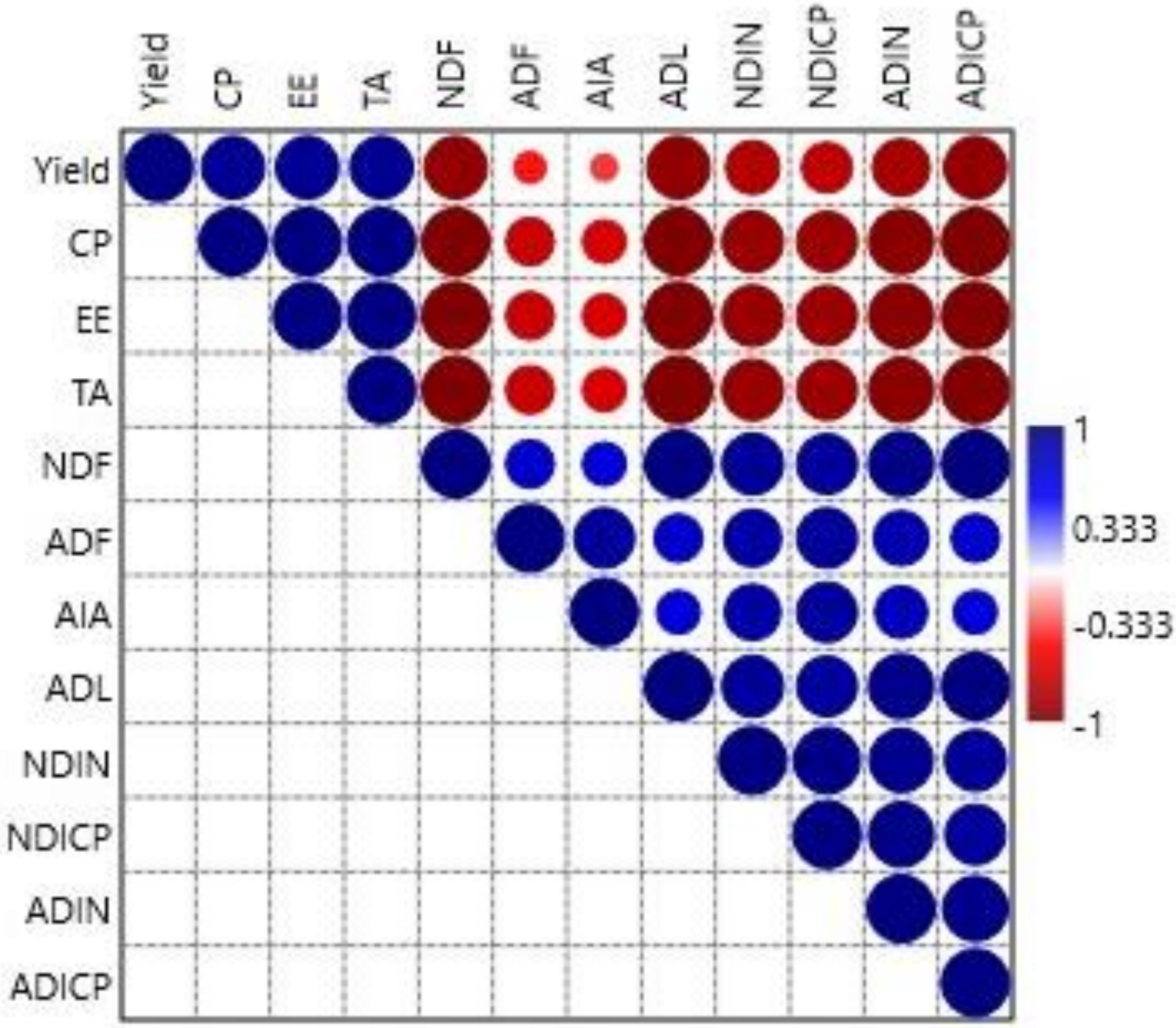

Suppl. Fig. 2B. Correlation plot matrix among yield, proximate composition and fiber fractions in maize during 2019.

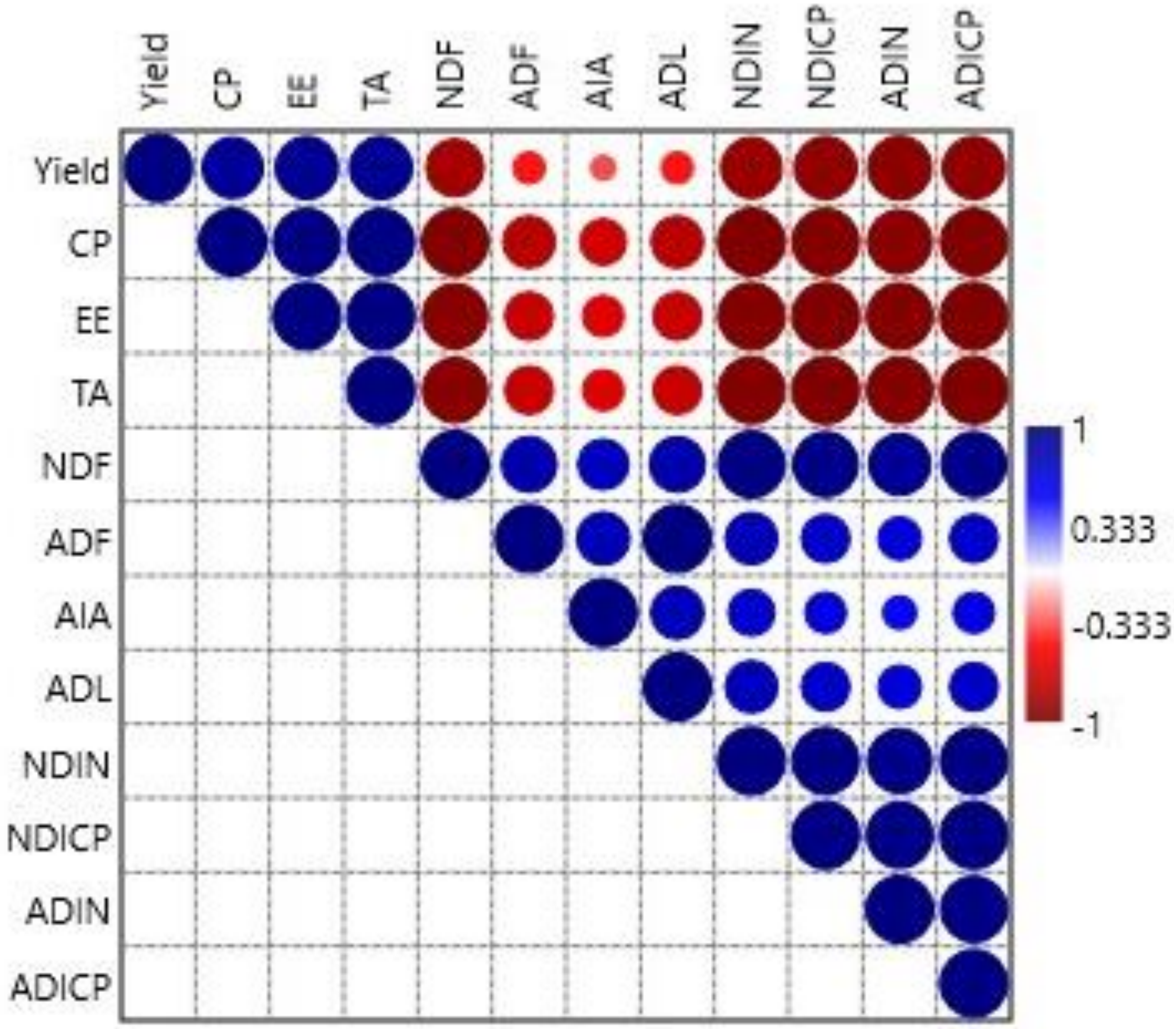

Suppl. Fig. 2C. Correlation plot matrix among yield, proximate composition and fiber fractions in maize during 2020.

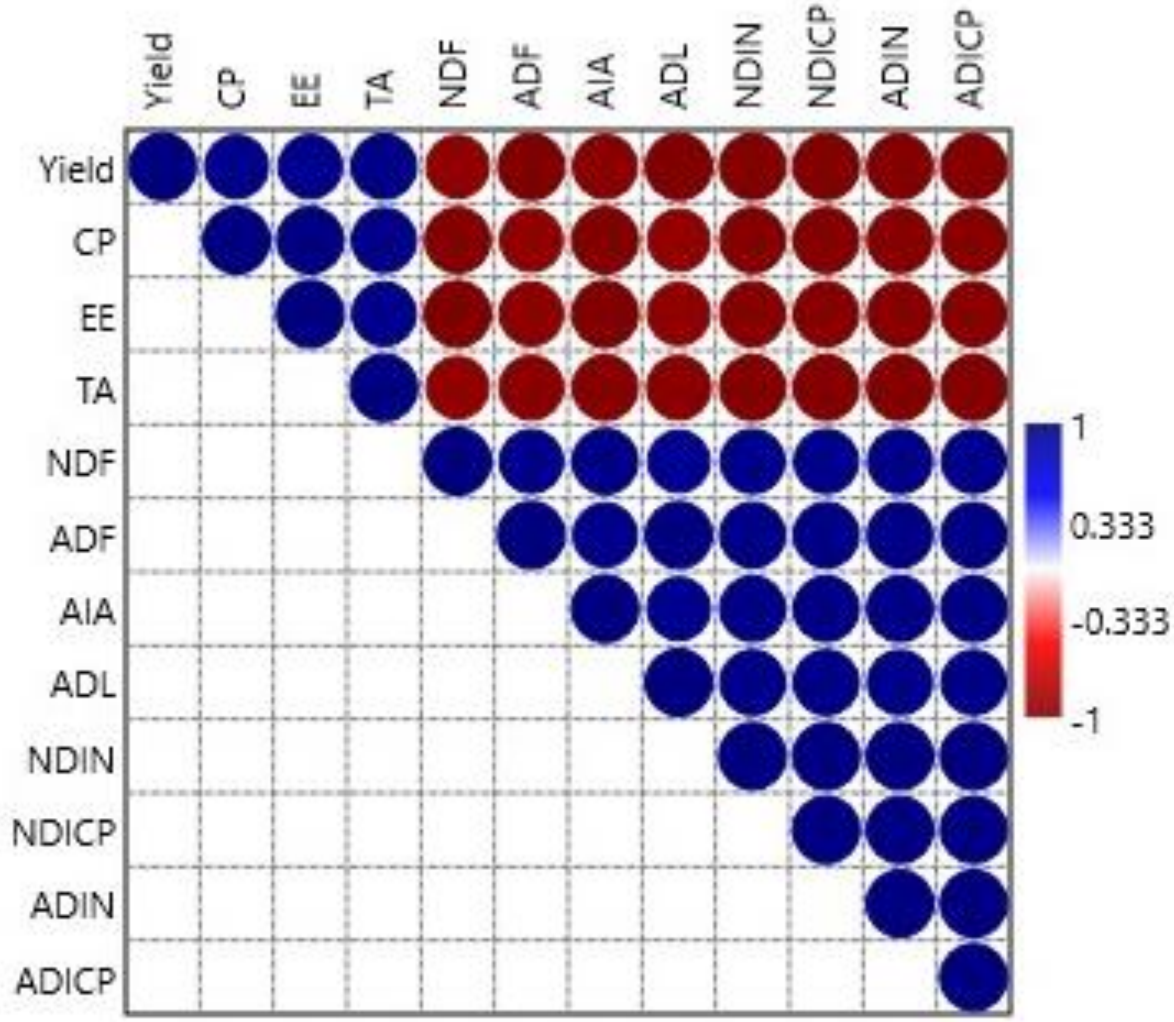

Suppl. Fig. 3A. Correlation plot matrix among yield, proximate composition and fiber fractions in berseem during 2018-19.

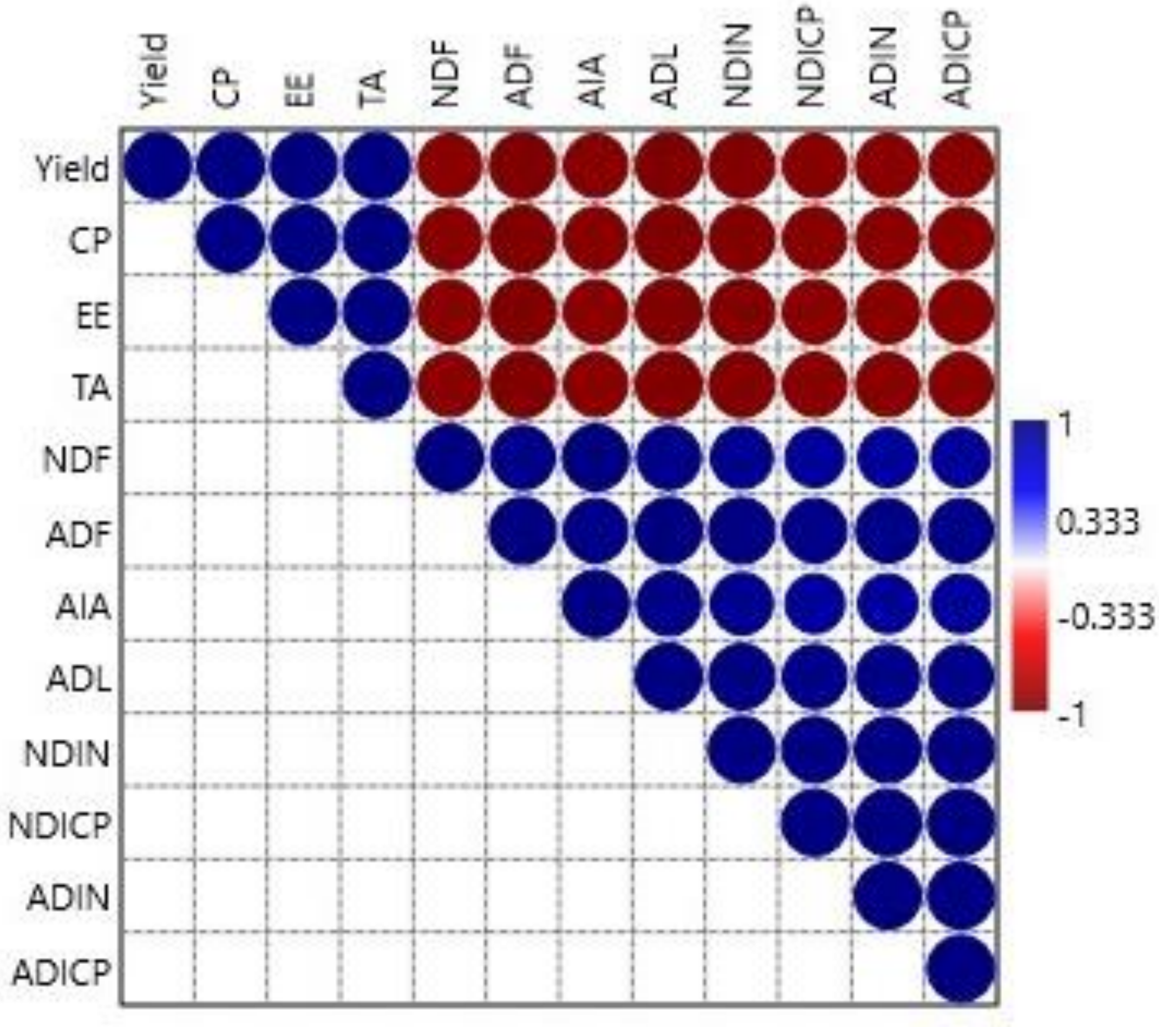

Suppl. Fig. 3B. Correlation plot matrix among yield, proximate composition and fiber fractions in berseem during 2019-20.

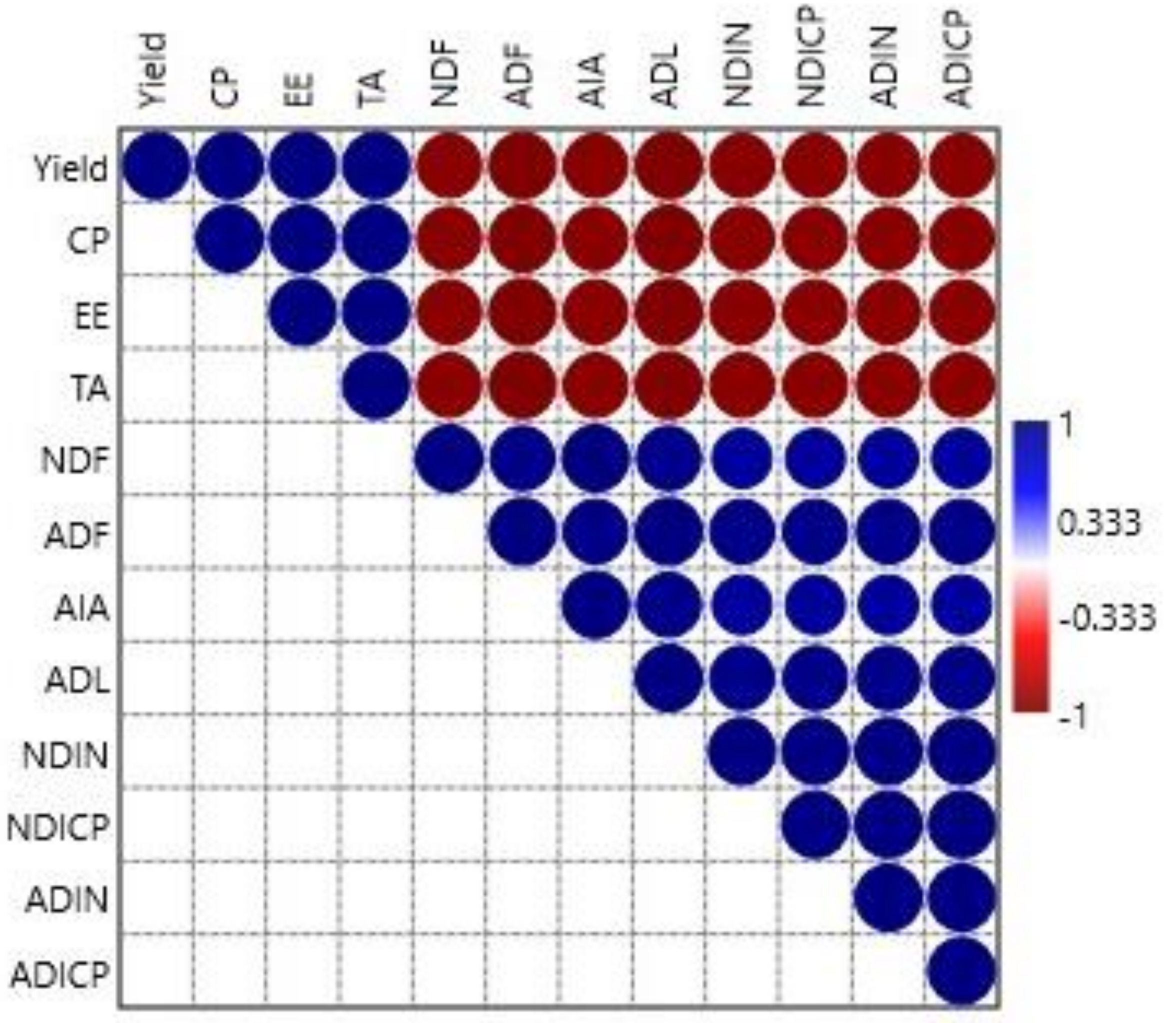

Suppl. Fig. 3C. Correlation plot matrix among yield, proximate composition and fiber fractions in berseem during 2020-21.

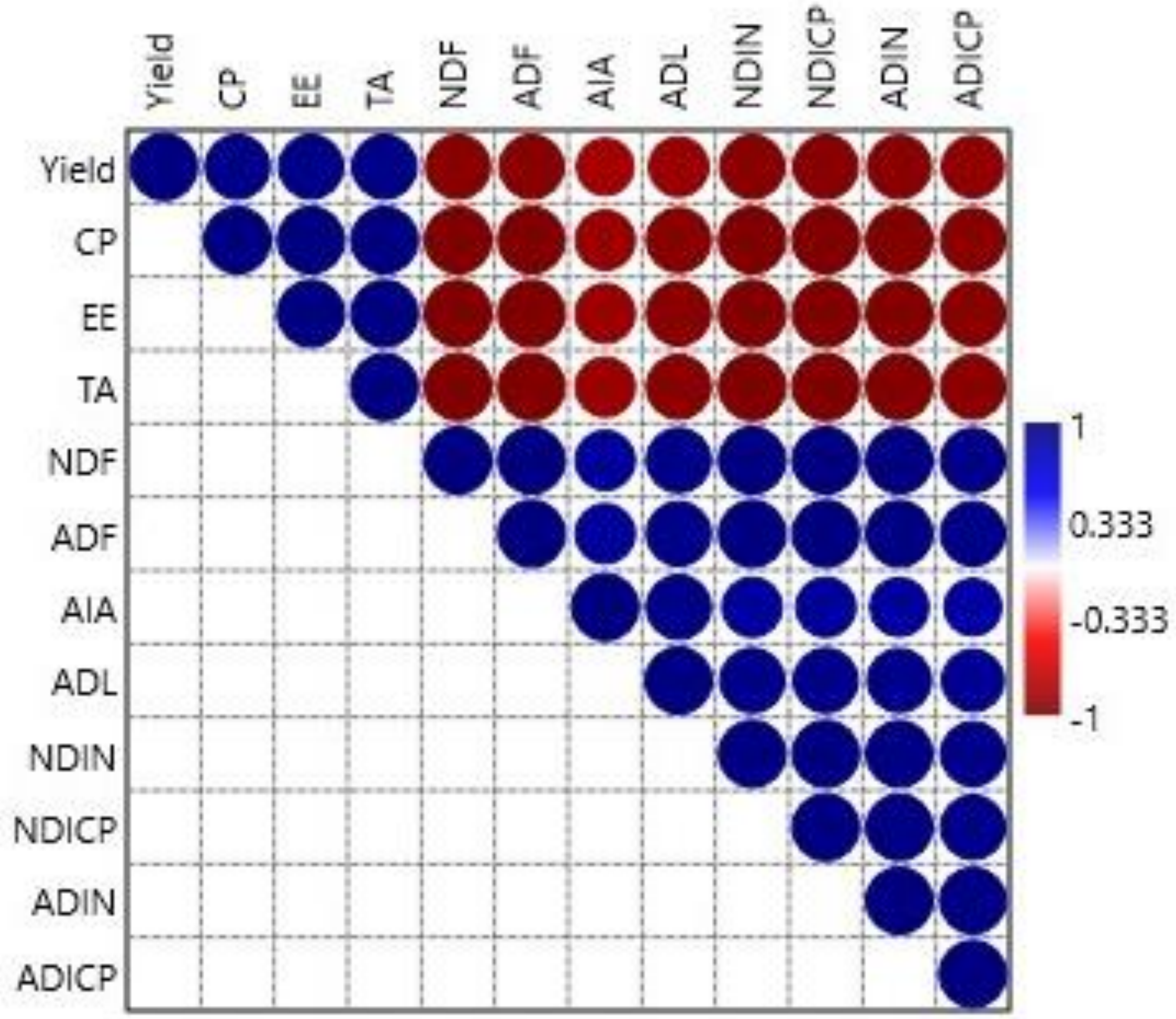

Suppl. Fig. 4A. Correlation plot matrix among yield, proximate composition and fiber fractions in cowpea during 2019.

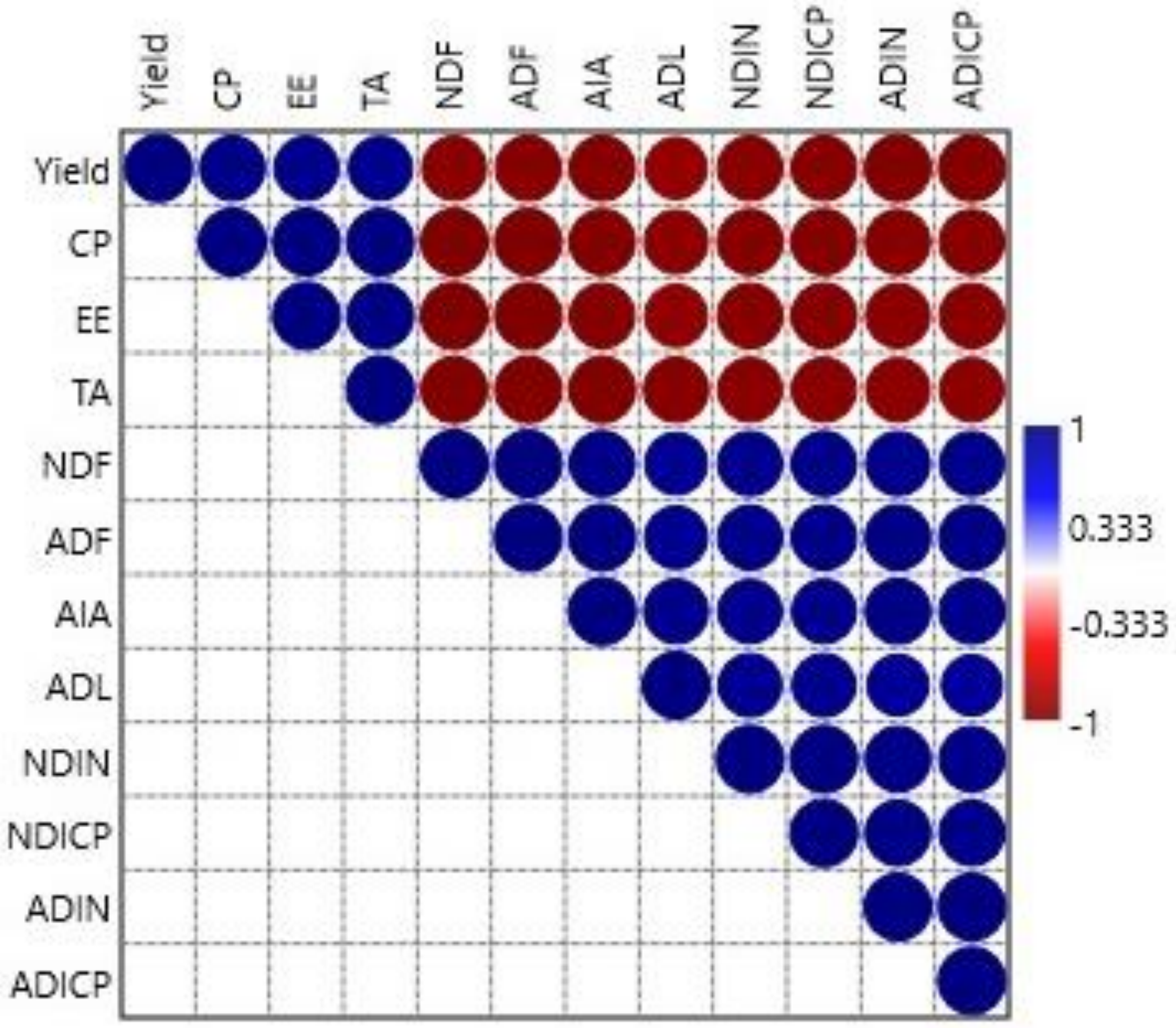

Suppl. Fig. 4B. Correlation plot matrix among yield, proximate composition and fiber fractions in cowpea during 2020.

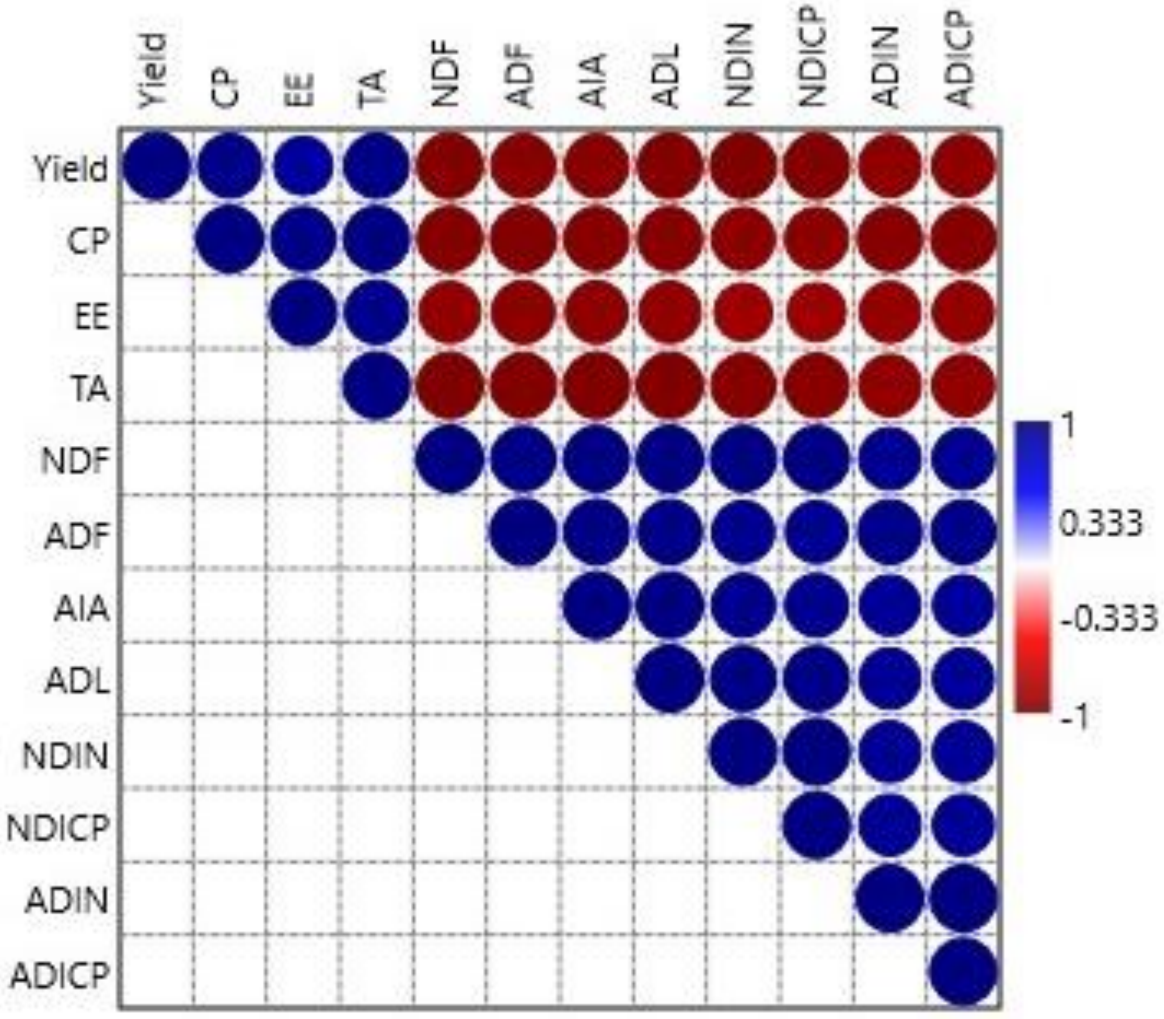

Suppl. Fig. 4C. Correlation plot matrix among yield, proximate composition and fiber fractions in cowpea during 2021.
